# Impact of medical insurance access negotiation on the utilization of innovative anticancer drugs in China: an interrupted time series analysis

**DOI:** 10.1186/s12913-023-10393-y

**Published:** 2024-01-17

**Authors:** Cui Li, Jingmin Zhu, Linghan Shan, Yingyu Zhou, Gang Liu, Hong Zhu, Qunhong Wu, Yu Cui, Zheng Kang

**Affiliations:** 1 Department of Social Medicine, School of Health Management, Harbin Medical University, Heilongjiang, China; 2 Department of Material management, Fourth Affiliated Hospital, Harbin Medical University, Heilongjiang, China; 3 Department of Epidemiology and Public Health, University College London, London, UK; 4 Centre for Health Economics and Policy Innovation, Department of Economics and Public Policy, Imperial Business School, London, UK; 5 Science and Technology Development Center of the Chinese Pharmaceutical Association, Peking, China; 6 Department of Acupuncture, Second Affiliated Hospital, Heilongjiang University of Chinese Medicine, Harbin, Heilongjiang China; 7 Department of Pharmaceutical Administration, School of Humanities and Social Sciences, Harbin Medical University, Heilongjiang, China

**Keywords:** Innovative anticancer drug, Medical insurance access negotiation, Drug expenditure, DDDs, Interrupted time series, China

## Abstract

**Background:**

The high costs of innovative anticancer drugs hinder a number of cancer patients’ access to these drugs in China. To address this problem, in 2018, the medical insurance access negotiation (MIAN) policy was implemented, when the prices of 17 innovative anticancer drugs were successfully negotiated and they were therefore included in the reimbursement list. This study aimed to explore the impact of the MIAN policy on the utilization of innovative anticancer drugs.

**Methods:**

With monthly data on drug expenditures and defined daily doses (DDDs) of each innovative anticancer drug from January 2017 to December 2019, interrupted time series analysis was employed to estimate both the instant (change in the level of outcome) and long-term (change in trends of outcomes) impacts of the MIAN policy on drug utilization in terms of drug expenditures and DDDs. Our sample consists of 12 innovative anticancer drugs.

**Results:**

From January 2017 to December 2019, the monthly drug expenditures and DDDs of 12 innovative anticancer drugs increased by about 573% (from US$8,931,809.30 to US$51,138,331.09) and 1400% (from 47,785 to 668,754), respectively. Overall, the implementation of the MIAN policy led to instant substantial increases of US$8,734,414 in drug expenditures and 158,192.5 in DDDs. Moreover, a sharper upward trend over time was reported, with increases of US$2,889,078 and 38,715.3 in the monthly growth rates of drug expenditures and DDDs, respectively. Regarding individual innovative anticancer drugs, the most prominent instant change and trend change in drug utilization were found for osimertinib, crizotinib, and ibrutinib. In contrast, the utilization of pegaspargase was barely affected by the MIAN policy.

**Conclusions:**

The MIAN policy has effectively promoted the utilization of innovative anticancer drugs. To ensure the continuity of the effects and eliminate differentiation, supplementary measures should be carried out, such as careful selection of drugs for medical insurance negotiations, a health technology assessment system and a multichannel financing mechanism.

**Supplementary Information:**

The online version contains supplementary material available at 10.1186/s12913-023-10393-y.

## Background

In 2020, an estimated 19.3 million new cancer cases and 10.0 million cancer deaths were reported worldwide, and China had 23.7% of new cases and 30% of deaths, ranking first worldwide [1]. To improve the quality of life of cancer patients and reduce the death rate, targeted therapy has recently been combined with cancer chemotherapy [2–4]. Despite reduced negative synergistic effects and better treatment effects, targeted drugs are usually expensive because of patent protection and supply-side monopolies [5–7]. According to the Global Oncology Trend 2019 [8], the oncology therapy market in China was worth approximately US$9 billion in 2018 and has more than doubled in the past five years. However, in anticancer drug spending of US$6.3 billion, innovative anticancer drugs (mainly targeted drugs), launched after 2013, comprised only 0.3% (US$218 million). In 2013, anticancer drug spending per capita in China amounted to US$4.50, compared to US$173 in the United States. This indicates the low accessibility and insufficient utilization of innovative anticancer drugs in China, mainly due to prohibitive prices and reimbursement restrictions from health insurance [9–11].

To address the problem of underutilization of drugs, different actions have been taken worldwide from the perspective of expanding medical insurance coverage. For example, in the UK, the National Institute of Health and Clinical Excellence (NICE) clarified the transformation mechanism of medical insurance access for patented drugs through legislation and introduced “value-based assessment” into the negotiation framework [12]. Canada implemented the Common Drug Review (CDR) system and set the medical insurance payment standard through the reference price of the Patent Drug Price Review Committee [13]. In Germany, after price negotiations conducted by the National Association of Statutory Health Insurance Funds (GKV-SV), drugs are listed in the medical insurance catalogue [14].

In China, before 2018, the bidding and procurement of drugs was presided over by the National Health Commission of China (NHC). Under this strategy, it was nearly impossible for the NHC to establish a complete contractual relationship with drug companies or implement volume bidding, resulting in inflated drug prices [15]. Aiming at reducing drug prices, a major reform was carried out on the bidding and procurement of expensive anticancer drugs in 2018 in China, including a new medical insurance access negotiation (MIAN) policy. The National Healthcare Security Administration (NHSA), which is in charge of national medical insurance funds, started to negotiate with drug companies as a real purchaser. The economic concept of “bulk purchasing” was also introduced in its price reduction strategy [15]. The drugs with agreed upon prices are included in the medical insurance reimbursement list. In November 2018, 17 innovative anticancer drugs were successfully negotiated and included in the medical insurance reimbursement list. To the best of our knowledge, only a few studies have examined the impact of the MIAN policy in China. For instance, Sun et al. [16] found that the MIAN policy promoted the use of 17 innovative anticancer drugs at Peking Union Medical College Hospital (a tertiary general public hospital) by improving their accessibility. Chen [17] found that at Peking University Cancer Hospital (a tertiary specialist hospital), the cancer patient burden was alleviated, while the rational use of clinical medicine was improved after the implementation of the MIAN policy. Cai et al. [18] found that the national drug price negotiation policy in 2018 improved the availability, utilization, and affordability of anticancer medicines. However, Xu et al. [19] reported that the economic burden on most cancer patients was still heavy in Tianjin city after the implementation of the MIAN policy. In addition, evaluations of similar policies have been conducted in other settings. Improved accessibility of drugs has not always been reported [9, 20, 21]. For example, Sruamsiri et al. [20] found that the E2 access program in Thailand, aimed at increasing access to high-cost medicines, has facilitated patients’ access to specialty medicines based on data from three hospitals. Aggarwal et al. [21] analysed the potential value of drugs approved by the Cancer Drugs Fund (CDF) in England and found that the CDF has not delivered meaningful value to NHS cancer patients. Hsu et al. [9] found that removing reimbursement restrictions for targeted drugs significantly increased the level and growth rate of drug accessibility in Taiwan based on claims data from 92,220 patients with non-small cell lung carcinoma.

Therefore, on the basis of previous studies, this study aimed to further evaluate the effect of the MIAN policy on the utilization of drugs nationwide in China and explore the difference between individual anticancer drugs. First, this study described the changes in the price and utilization, which was measured by expenditures and defined daily doses (DDDs), for each negotiated anticancer drug. Second, interrupted time series analysis was adopted to evaluate the effect of the MIAN policy on drug utilization, together and seperately. Finally, based on our findings, we discussed the outcomes of the MIAN policy and provided policy implications. Compared to the similar study of Cai and colleagues, our study provided a more comprehensive picture of the impact of MIAN policy on the utilisation of innovative anticancer drugs by showing price changes as a direct outcome of the MIAN policy and including monthly drug expenditure as an additional measure of utilization. We also contributed to the literature with a detailed discussion on varied changes in individual drugs, which enabled us to provide more explicit implications for policy makers.

## Methods

### Data source

Data were obtained from the China Medical Economic Information Network (CMEI), which was founded by the Science and Technology Development Center of the Chinese Pharmaceutical Association in 1993 [22]. The CMEI has set up 35 subnetworks in China with more than a thousand network members across the country, covering all administrative areas except Hong Kong, Macao, and Taiwan. 55% of tertiary hospitals in China are in the CMEI. The CMEI has gradually developed into the most extensive, sustained, and authoritative medical information service platform in China.

Monthly data of 17 innovative anticancer drugs from January 2017 to December 2019 were collected from 1,027 hospitals in the CMEI. As shown in Table S1, there were 739 tertiary hospitals and 288 secondary hospitals, i.e., 748 general hospitals, 185 specialized hospitals, and 49 other types of hospitals (traditional Chinese medical hospitals included). Retrieved data included the drug’s generic name, dosage form, specification, price, ATC code, expenditure, amount, etc.

### Study sample

There were 17 innovative anticancer drugs listed after the MIAN policy. According to the Anatomical Therapeutic Chemical classification system, these drugs are classified into five categories: pyrimidine analogues (L01BC), monoclonal antibodies (L01XC), protein kinase inhibitors (L01XE), other antineoplastic agents (L01XX), and antigrowth fibrinoids (H01CB). Detailed information on generic names, ATC codes, doses, and prices before and after the policy is provided in the supplementary materials (Table S2). Five of the 17 anticancer drugs were excluded from our sample since they were only introduced to Chinese market in late 2017 or 2018: azacitidine, anlotinib, ceritinib, vemurafenib, and ixazomib. Therefore, our sample consists of 12 anticancer drugs.

### Outcome variables

Drug utilization was measured by drug expenditures and DDDs. Drug expenditure is a generic term (including all specifications and models) considered as the standard to calculate the monthly total expenditure of drug utilization of an anticancer drug. Drug expenditure was recorded in Chinese Yuan (CNY) and converted to US dollars.^1^ DDDs is a measurement of drug consumption quantity independent of price, package size or strength, reflecting the medication dynamics and structure [23, 24]. The greater the DDDs, the higher the frequency of drug use. The DDDs is calculated as the ratio of the consumption volume of a drug to the defined daily dose (DDD) over a month. The DDD is recommended by the WHO and Pharmacopoeia of the People’s Republic of China and the New Materia Medica.

$${\text{DDDs}} = ({\text{consumption volume}})/{\text{DDD}}$$

### Statistical analysis

Since the 1970s, interrupted time series (ITS) analysis has been used to evaluate the effect of public policy intervention [25]. At present, ITS is considered to be the strongest quasi-experimental research design for evaluating the longitudinal effects of policy interventions [26, 27], with the advantage of controlling for and excluding the influence of other historical or mature factors on the long-term trend and explain the short-term and long-term effects and delays of policy effects [28]. In this study, ITS was employed to estimate both the transient (the instant change in outcomes) and long-term (the change in trends of outcomes) effects of the MIAN policy on drug utilization. The basic model was as follows.

$$\begin{aligned} {{\text{Y}}_{\text{t}}} & = {\beta _0} + {\beta _1} \times {\text{tim}}{{\text{e}}_{\text{t}}} + {\beta _2} \times {\text{interventio}}{{\text{n}}_{\text{t}}} + {\beta _3} \\ & \quad \times {\text{time}}\_{\text{after}}\_{\text{interventio}}{{\text{n}}_{\text{t}}} + {{\text{e}}_{\text{t}}} \\ \end{aligned}$$

where Y_t_ is the drug expenditure or DDDs in month t; time_t_ is a continuous variable indicating time in months from the start of the observation period; intervention_t_ equals 1 from November 2018 onwards and 0 otherwise; and time_after_intervention_t_ is a continuous variable indicating months passed since the intervention (time prior to the intervention is coded 0). Immediate changes in the level of outcomes after the MIAN policy are indicated by β_2_, and changes in the trend after the MIAN policy are reported by β_3_ [24, 29]. Our models also controlled for autocorrelation [30]. To identify the most parsimonious models, we used backward elimination and excluded nonsignificant terms (p > 0.05). All analyses were conducted in R (R Core Team, 2022) and figures were produced using the package ggplot2 (Wickham, 2009).

## Results

### Descriptive statistics

**Fig. 1:**
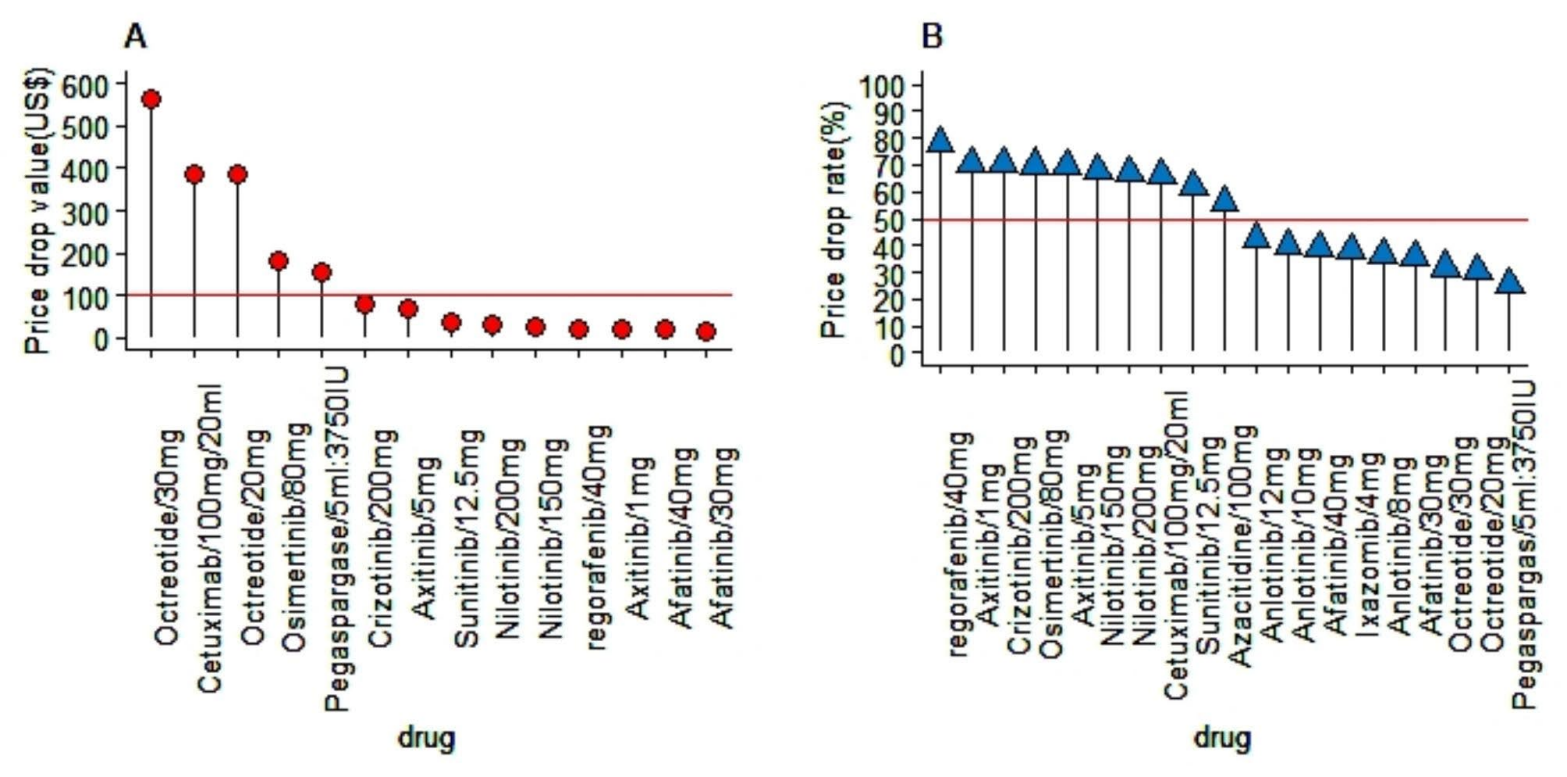
Price drop in value and rate for ten innovative anticancer drugs. *Notes*: Only ten drugs are included because the price of pazopanib and ibrutinib before the MIAN policy was not available. Each of the four drugs, i.e., octreotide, nilotinib, afatinib, and axitinib, has two different specifications. Left: value in US$ of drug price drop. Right: percentage change of price drop

As shown in Fig. 1, after the implementation of the MIAN policy, the average drop in the price of ten innovative anticancer drugs was US$141.96, with the smallest drop of US$13.73 and the largest of US$561.17. Five drugs saw price declines of more than US$100. The average decrease rate in the price was 53.66%, with the lowest decrease rate being 25.54% and the highest being 70.70%. Over half of the drugs had a decrease rate of more than 50%.

### Drug expenditures

**Fig. 2:**
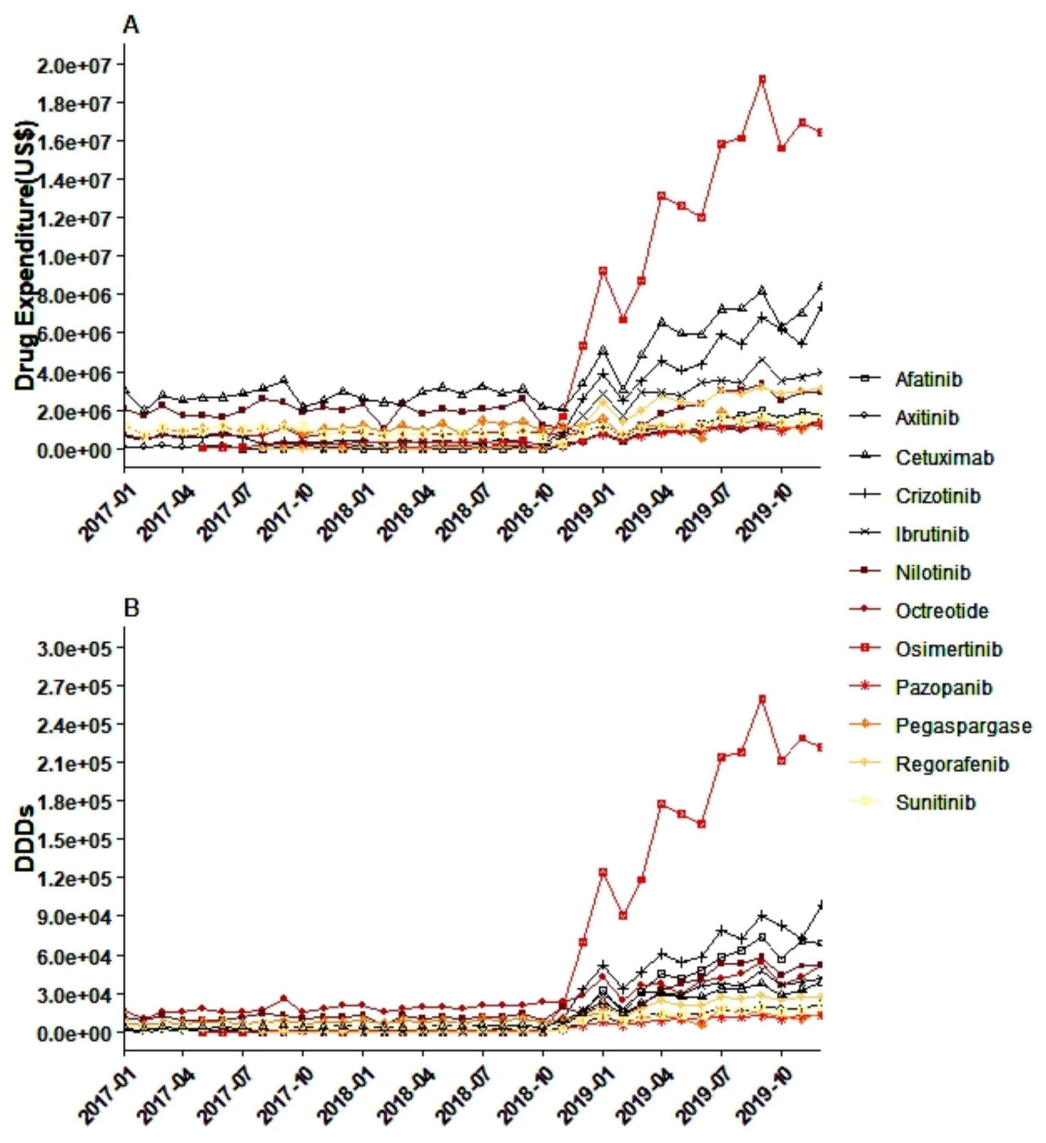
Drug expenditures (**A**) and DDDs (**B**) of the 12 innovative anticancer drugs from 2017 to 2019

Figure 2 depicts the monthly expenditures for the 12 innovative anticancer drugs from January 2017 to December 2019. Before the MIAN policy, drug expenditures were flat. After November 2018, rapid surges were observed in three drugs. Compared with before November 2018, the total expenditure of the 12 innovative anticancer drugs increased by an average of US$3,491,266.32 in December 2019. Osimertinib had the largest increase (US$14,731,617.63), followed by crizotinib (US$6,557,418.35) and cetuximab (US$6,338,749.00), and the smallest increase of US$216,813.82 was observed for pegaspargase. However, the highest average growth rate was for afatinib (24.04%), followed by osimertinib (19.2%), regorafenib (18.64%), and crizotinib (18.4%).

### DDDs

According to the monthly trends of the 12 innovative anticancer drugs (Fig. 2), the DDDs of all drugs increased after November 2018, especially osimertinib and crizotinib, with increases of 200,850 and 88,321, respectively. The average growth rate of the 12 innovative anticancer drugs was 12.74%. The highest were for afatinib (24.13%), osimertinib (18.94%), regorafenib (18.02%), crizotinib (17.7%), and Pazopanib (15.43%).

### Interrupted time series analysis

**Fig. 3:**
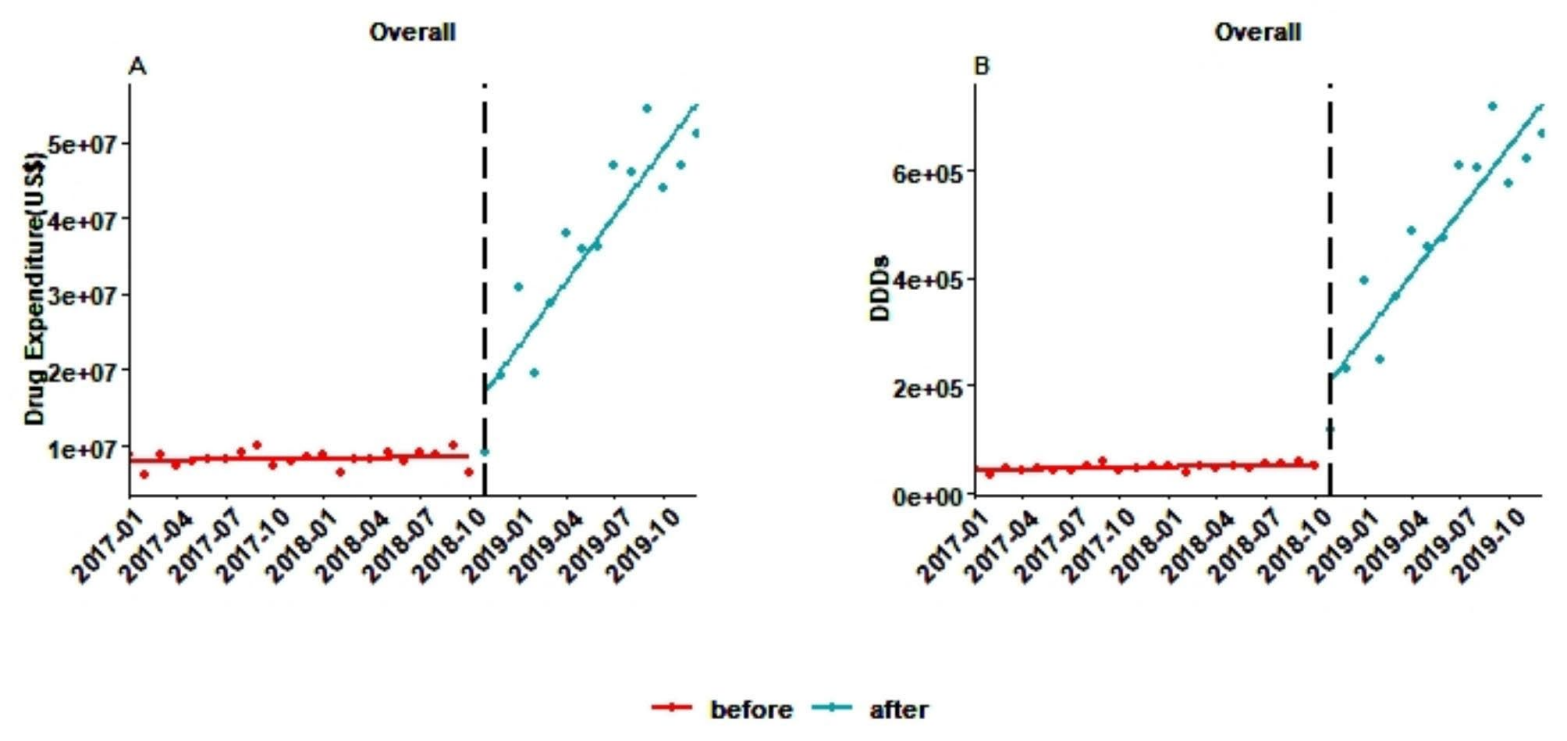
Trend in monthly overall expenditure (**A**) and DDDs (**B**) of 12 anticancer drugs from 2017 to 2019

The ITS analysis results on the effect of the MIAN policy on the expenditures and DDDs of 12 innovative anticancer drugs are reported in Table 1. Figure 3 depicts trend in overall monthly expenditure and DDDs after the ITS analysis. Separate trends of 12 anticancer drugs are reported in supplementary materials Figs. S1 and S2.

### Drug expenditures

Before the MIAN policy was launched, there was no significant monthly change in the expenditures of any drugs. With the implementation of the MIAN policy, significant instant changes occurred to eight of 12 drugs, including increases in the expenditure of seven drugs: afatinib (US$323,383), axitinib (US$291,255), osimertinib (US$4,514,591), crizotinib (US$1,625,733), pazopanib (US$250,417), regorafenib (US$1,204,753), and ibrutinib (US$1,530,797), and a decrease in the expenditure of nilotinib (US$-1,086,056). The overall increase in drug expenditure was US$8,734,414. In addition, 11 drugs except pegaspargase experienced a significant upward trend. The overall significant increase in the growth rate of drug expenditures was US$2,889,078, as shown in Fig. 3A as well.

### DDDs

Similarly, before the MIAN policy, the monthly change in DDDs was not significant. A significant instant increase was noted after the implementation of the MIAN policy for nine drugs, including afatinib (11,615), axitinib (5,653), osimertinib (63,063), crizotinib (23,887), pazopanib (3,619), regorafenib (10,590), sunitinib (4,148), and ibrutinib (56,800). There were statistically significant increases in the trend of 11 drug DDDs after the MIAN policy except pegaspargase. In Fig. 3B, there was a rapid rise shortly and faster steady growth in the long term after the introduction of the MIAN policy.

**Table 1:** Results of interrupted time series analysis of 12 innovative anticancer drugs

Drug	Drug expenditures (US$)					DDDs			
	Intercept β0	Baseline trend β1	Level change after MIAN β2	Trend change after MIAN β3		Intercept β0	Baseline trend β1	Level change after MIAN β2	Trend change after MIAN β3
Afatinib	8,500	-115.74	323,383^**^	134,647^***^		172.98	-1.93	11,615^**^	5,044^***^
Axitinib	86,661	-523.95	291,255^***^	73,488^***^		345.76	12.68	5,653^***^	1,201^***^
Octreotide	755,307	7,284	-70,982	36,415^***^		17,411	336.78	5,525	1,301^**^
Osimertinib	291,844	3,160	4,514,591^***^	1,112,752^***^		1,021	30.94	63,063^***^	15,180^***^
Crizotinib	375,945	-4,083	1,625,733^***^	403,016^***^		1,228	28.43	23,887^***^	5,347^***^
Nilotinib	2,029,676	-11,501	-1,086,056^***^	209,580^***^		10,766	6.73	2,790	3,486^***^
Pegaspargase	1,034,835	14,284	-113,896	-36.85		7,454	121.10	1,697	55.08
Pazopanib	7,859	10,096	250,417^**^	58,425^***^		-28.50	42.69	3,619^***^	701.62^***^
Regorafenib	18,490	1,094	1,204,753^***^	168,215^***^		76.36	7.10	10,590^***^	1,497^***^
Sunitinib	804,672	-6,059	-79,592	92,918^***^		3,180	-0.83	4,148^***^	952.59^***^
Cetuximab	2,653,678	16,055	321,573	387,434^***^		3,642	87.10	9,586^***^	1,792^***^
Ibrutinib	18,644	4,075	1,530,797^***^	207,407^***^		88.64	71.75	56,800^***^	7,718^***^
Overall	8,155,256	28,948	8,734,414^**^	2,889,078^***^		37,480.70	739.50	158,192.50^***^	38,715.30^***^

*Note*: ***p < 0.01, **p < 0.05

## Discussion

This study examined the impact of the MIAN policy implemented in November 2018 on the utilization of 12 innovative anticancer drugs. With monthly data of 12 innovative anticancer drug from January 2017 to December 2019 collected from 1,027 hospitals in China, our results suggested that the MIAN policy increased the utilization of anticancer drugs. Both the overall drug expenditures and DDDs showed a sharp instant increase, and a higher growth rate after the implementation of the MIAN policy.

Our findings are consistent with existing evidence on the effectiveness of drug policies in promoting drug utilization in different contexts [9, 18, 20, 31, 32]. For example, the study by Hsu et al. [9] regarding the suspension of reimbursement restrictions on targeted drugs in Taiwan reported that the level and growth rate of targeted drug accessibility were increased. Sruamsiri et al. mentioned that the high-cost medicine E2 access program facilitated patients’ access to high-cost specialty medicines and decreased treatment costs in Thailand [20]. The study of Sun et al. reported that the overall cost of antidiabetic drugs was immediately reduced by 4.4 billion KRW in the month after the implementation of a national price cut program in Korea [31]. Stephens P et al. confirmed that the CDF in England has shortened the waiting time for cancer patients to receive innovative treatments and greatly reduced the burden on patients [32]. With the focus on the MIAN policy as well, Cai et al. evaluated its impact on drug utilization measured by DDDs and found that the MAIN policy improved the overall utilization of anticancer drugs both immediately and thereafter [18]. Their results on the utilization of cetuximab were also consistent with ours [18].

In addition, changes in the utilization of the 12 anticancer drugs differed greatly, in particular, as osimertinib, crizotinib and ibrutinib experienced the most outstanding increases, whilst pegaspargase had neglectable change. It might be explained by varied drug properties, the availability of alternative drugs and cancer incidence in China.

To be more specific, osimertinib, as a third-generation epidermal growth factor receptor (EGFR) inhibitor, is a first-line treatment for EGFR-positive advanced non-small cell lung cancer [33]. It can effectively counter T790M mutations and EGFR-sensitive mutations, and overcome first-generation EGFR-TKI drug resistance and selectivity [34]. Therefore, in terms of drug properties, osimertinib has an absolute advantage over other non-small cell anticancer drugs. In addition, China has a large number of lung cancer patients, and the annual incidence rate of lung cancer is no less than 57.26 per 100,000 [35]. It is therefore plausible that when the price of osimertinib fell by 70%, its utilization increased greatly.

Crizotinib is the first approved anaplastic lymphoma kinase-tyrosine kinase inhibitors (ALK-TKIs) and is the first-line drug recommendation for patients with ALK-positive NSCLC [36]. With an objective response rate of 60% in ALK-positive non-small cell lung cancer and a progression-free survival of 7 to 10 months, it is superior to standard first-line pemetrexed plus platinum chemotherapy and has an advantage of clinical validation [37]. As its price was reduced under the MIAN’s policy, the utilization of crizotinib soared.

Bruton tyrosine kinase (BTK) is a therapeutic target for B cell associated tumors. Ibrutinib, as the world’s first BTK inhibitor, has brought a breakthrough in the efficacy of B cell malignancies (BCM) [38]. Ibrutinib has been widely used in China without alternatives until June 2020. Hence its utilization is large, especially after the MIAN policy.

Pegaspargase is used as a treatment in children with acute lymphoblastic leukaemia (ALL), with its event-free survival rate as high as almost 80% in 2007 [39–41]. However, its alternative drugs (L-asparaginase), which were approved 40 years earlier, have nearly the same efficacy and adverse effects and higher reimbursement rate in medical insurance [16, 42]. Therefore, the utilization of pegaspargase was barely affected by the MIAN policy. The lower incidence rate of ALL might also explain no significant changes in utilization. According to the White Paper on Pediatric Hematology in China 2020, the incidence of ALL among children aged 0–14 years in China was 24.9 per 100,000 [43], lower than that of other types of cancers. In addition, the lack of advertisement for pegaspargase even after the MIAN policy might have contributed to the unpronounced change in its utilization as well.

Moreover, octreotide, sunitinib and nilotinib showed an immediate decrease in drug expenditures. This was mainly related to the fact that the use of octreotide, sunitinib and nilotinib increased less than their price decline. With the release of the MIAN policy dividend, when the increase in the use of these drugs exceeded the decrease in their prices, the expenditures of these drugs gradually increased.

Our study has two notable strengths. On the one hand, our data cover as many as 1,024 hospitals across the country, with the exception of Tibet. This makes our results roughly nationally representative. On the other hand, the impact of the MIAN policy is examined on each anticancer drug in detail. The differences in the utilization of the 12 innovative anticancer drugs are explained in light of the attributes of anticancer drugs, alternative drugs and cancer incidence in China, which provides references for policy making and for enterprises to research and develop drugs [44].

However, the results of this study should be interpreted in light of several limitations. First, we were unable to assess the drug utilization of individual patients due to data availability. Drug utilization data provide only an estimate of the volume of medications consumed and do not present a precise picture of actual use. Second, we included in our sample only 12 innovative anticancer drugs affected by the MIAN policy, which did not represent all anticancer drugs. Finally, although the utilization of drugs has increased through changes in health insurance policies, health care resource allocation and health inequalities between various cancer types or diseases need further research.

## Conclusions

In 2018, the MIAN policy was implemented in China to reduce the high price of innovative anticancer drugs. After the MIAN policy, the drug expenditures and DDDs of most anticancer drugs increased, indicating that the policy effectively promoted the utilization of anticancer drugs. Among all the 12 anticancer drugs in our sample, the greatest increase was observed in the utilization of osimertinib, crizotinib, and ibrutinib, whilst the utilization of pegaspargase didn’t change significantly.

Several policy implications are provided. First, in the selection of anticancer drugs for medical insurance negotiations, the attributes of anticancer drugs and cancer incidence should be considered to predict the effectiveness of intervention and benefit more cancer patients. Second, a health technology assessment system should be established [45–48], to estimate the economic benefit and clinical value of drugs and to evaluate the access and withdrawal of drugs. Lastly, in order to cope with rising innovative anticancer drugs expenditures, apart from medical insurance negotiations, a multichannel financing mechanism is necessary and the efficiency of fund use needs to be improved.

### Electronic supplementary material

Below is the link to the electronic supplementary material.

Supplementary Material 1: Brief profile of sample hospitals and changes in individual anti-cancer drug price, expenditure and DDDs

## Data Availability

All data were treated as confidential and not publicly available but could be disclosed through the correspondence author on a reasonable request.

## Footnotes

1 The annual average exchange rates were 6.7518, 6.6174, and 6.8985 CNY per US dollar in 2017, 2018, and 2019, respectively, according to the Statistical Bulletin of National Economic and Social Development of the People’s Republic of China.

## Abbreviations

MIAN: Medical Insurance Access Negotiation
DDDs: Defined Daily Doses
NICE: National Institute of Health and Clinical Excellence
CDR: Common Drug Review
NHC: National Health Commission of China
NHSA: National Healthcare Security Administration
CDF: Cancer Drugs Fund
CMEI: China Medical Economic Information
CNY: Chinese Yuan
ITS: Interrupted time series
EGFR: Epidermal growth factor receptor
RCC: Renal cell carcinoma
HR-QOL: Health-related quality of life
