# Supplementary material for: Impact of medical insurance access negotiation on the utilization of innovative anticancer drugs in China: an interrupted time series analysis
Source: BMC Health Serv Res. 2024 Jan 17;24:90. doi: 10.1186/s12913-023-10393-y (PMC10792910; doi:10.1186/s12913-023-10393-y)
Supplement: Supplementary file 1 — Supplementary Material 1: Brief profile of sample hospitals and changes in individual anti-cancer drug price, expenditure and DDDs [file 12913_2023_10393_MOESM1_ESM.docx]

**Supplementary file 1. Brief profile of sample hospitals and changes in individual anti-cancer drug price, expenditure and DDDs**

**Table S1. Brief profile of sample hospitals**

| **Hospital Characteristics** | **Number** | **Proportion** |
| --- | --- | --- |
| By Hospital Level |  |  |
| Secondary | 288 | 28.04% |
| Tertiary | 739 | 71.96% |
| By Types of Hospitals |  |  |
| General | 748 | 72.83% |
| Specialized | 185 | 18.01% |
| Else (TCM* included) | 94 | 9.16% |

*TCM: Traditional Chinese Medical hospital.

Note: Hospitals in Tibet are excluded.

**Table S2. Price comparison before and after anti-cancer drug medical insurance access negotiations**

| **Generic name** | **ATC Code** | **Dose** | **pre-MIAN price ($)** | **post-MIAN price ($)** |
| --- | --- | --- | --- | --- |
| Cetuximab | L01XC | 100 mg/20 ml inj | 581.11 | 195.70 |
| Afatinib | L01XE | 40 mg tab | 49.41 | 30.22 |
|  |  | 30 mg tab | 37.98 | 24.25 |
| Axitinib | L01XE | 5 mg tab | 102.01 | 31.28 |
|  |  | 1 mg tab | 31.16 | 9.13 |
| Osimertinib | L01XE | 80 mg tab | 257.76 | 77.07 |
|  |  | 40 mg tab | - | 45.34 |
| Crizotinib | L01XE | 250 mg cap | - | 39.29 |
|  |  | 200 mg cap | 112.81 | 33.12 |
| Nilotinib | L01XE | 200 mg cap | 43.90 | 14.31 |
|  |  | 150 mg cap | 35.96 | 11.48 |
| Pazopanib | L01XE | 400 mg tab | - | 41.10 |
|  |  | 200 mg tab | - | 24.18 |
| regorafenib | L01XE | 40 mg tab | 52.70 | 29.62 |
| Sunitinib | L01XE | 50 mg cap | - | 67.70 |
|  |  | 37.5 mg cap | - | 54.31 |
|  |  | 25 mg cap | - | 39.82 |
|  |  | 12.5 mg cap | 61.51 | 23.42 |
| Ibrutinib | L01XE | 140 mg cap | - | 28.56 |
| Pegaspargas | L01XX | 5 ml:3750 IU inj | 604.77 | 450.33 |
|  |  | 2 ml:1500IU inj | - | 223.31 |
| Octreotide | H01CB | 30 mg bot | 1756.65 | 1195.48 |
|  |  | 20 mg bot | 1261.61 | 876.48 |

**Table S3 Drug expenditures of the 12 innovative anticancer drugs from 2017 to 2019**

| **Generic name** | **2017H1** | **2017H2** | **2018H1** | **2018H2** | **2019H2** | **2019H2** |
| --- | --- | --- | --- | --- | --- | --- |
| Afatinib |  | 27,221.48 | 57,635.93 | 551,234.93 | 5,793,239.98 | 10,545,874.47 |
| Axitinib | 636,896.83 | 677,605.97 | 478,895.03 | 977,386.13 | 4,358,611.44 | 6,847,150.97 |
| Octreotide | 3,981,893.27 | 4,702,564.50 | 4,608,223.92 | 4,915,332.00 | 5,848,908.89 | 7,526,570.56 |
| Osimertinib | 77,934.77 | 1,142,017.68 | 1,953,939.61 | 8,314,398.40 | 62,341,317.68 | 100,036,220.92 |
| Crizotinib | 3,896,794.93 | 2,276,246.33 | 2,117,323.72 | 4,745,615.95 | 22,936,251.07 | 37,225,431.62 |
| Nilotinib | 10,954,851.74 | 12,971,308.84 | 11,390,069.36 | 9,831,984.77 | 9,413,995.22 | 17,791,147.64 |
| Pegaspargase | 6,017,154.68 | 6,074,684.82 | 6,382,370.87 | 7,344,006.56 | 6,171,866.49 | 8,323,033.99 |
| Pazopanib |  | 10,219.50 | 408,740.59 | 992,293.05 | 4,516,576.07 | 6,586,684.06 |
| Regorafenib |  | 84,222.73 | 169,386.77 | 1,655,237.71 | 13,199,719.94 | 17,984,353.12 |
| Sunitinib | 5,969,473.47 | 5,884,885.07 | 4,601,513.43 | 4,087,670.69 | 6,491,912.73 | 9,373,202.87 |
| Cetuximab | 15,562,446.01 | 17,170,377.97 | 16,245,102.61 | 16,783,709.16 | 31,437,422.63 | 44,417,129.67 |
| Ibrutinib |  | 7,198.08 | 281,726.36 | 2,610,155.05 | 16,560,000.00 | 22,734,246.58 |
| Overall | 47,097,445.72 | 51,028,552.98 | 48,694,928.22 | 62,809,024.39 | 189,069,822.14 | 289,391,046.46 |

Notes: The expenditures of afatinib, pazopanib, regorafenib, and ibrutinib were not available in 2017H1.

**Table S4 Drug DDDs of the 12 innovative anticancer drugs from 2017 to 2019**

| **Generic name** | **2017H1** | **2017H2** | **2018H1** | **2018H2** | **2019H1** | **2019H2** |
| --- | --- | --- | --- | --- | --- | --- |
| Afatinib |  | 581 | 1,190 | 19,194 | 213,439 | 391,361 |
| Axitinib | 3,052 | 3,248 | 2,254 | 12,457 | 72,506 | 113,855 |
| Octreotide | 89,157 | 110,600 | 111,271 | 137,215 | 209,700 | 270,286 |
| Osimertinib | 300 | 4,380 | 7,350 | 95,970 | 843,258 | 1,353,137 |
| Crizotinib | 14,790 | 8,640 | 7,950 | 49,950 | 305,536 | 496,623 |
| Nilotinib | 60,691 | 71,739 | 61,818 | 77,912 | 164,603 | 311,288 |
| Pegaspargase | 42,258 | 43,602 | 46,119 | 58,942 | 61,569 | 83,005 |
| Pazopanib |  | 38 | 1,472 | 7,561 | 48,685 | 70,998 |
| regorafenib |  | 392 | 777 | 13,615 | 116,146 | 158,247 |
| Sunitinib | 24,311 | 24,108 | 18,732 | 25,452 | 72,219 | 104,282 |
| Cetuximab | 22,648 | 25,382 | 23,912 | 42,401 | 144,280 | 203,849 |
| Ibrutinib |  | 26 | 1,002 | 24,918 | 172,697 | 237,087 |

Notes: The DDDs of afatinib, pazopanib, regorafenib, and ibrutinib were not available in 2017H1.


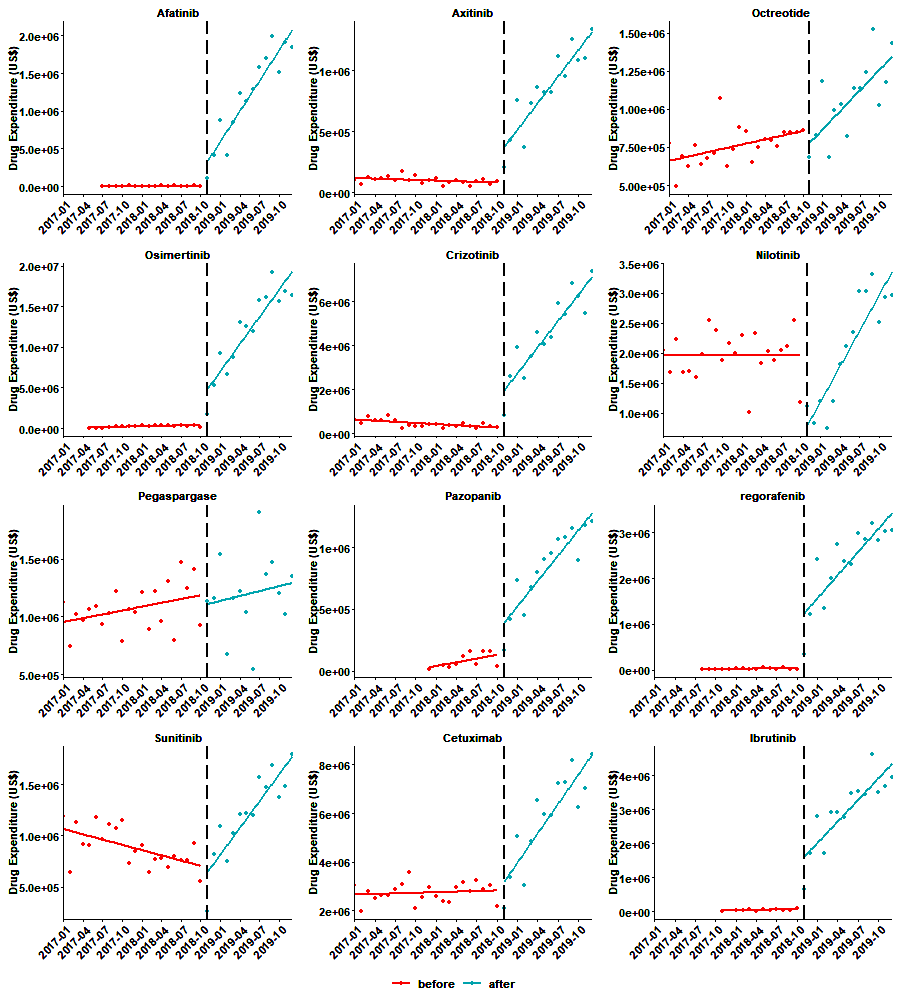


**Figure S1. Trend in monthly expenditures of 12 anticancer drug from January 2017 to December 2019.**


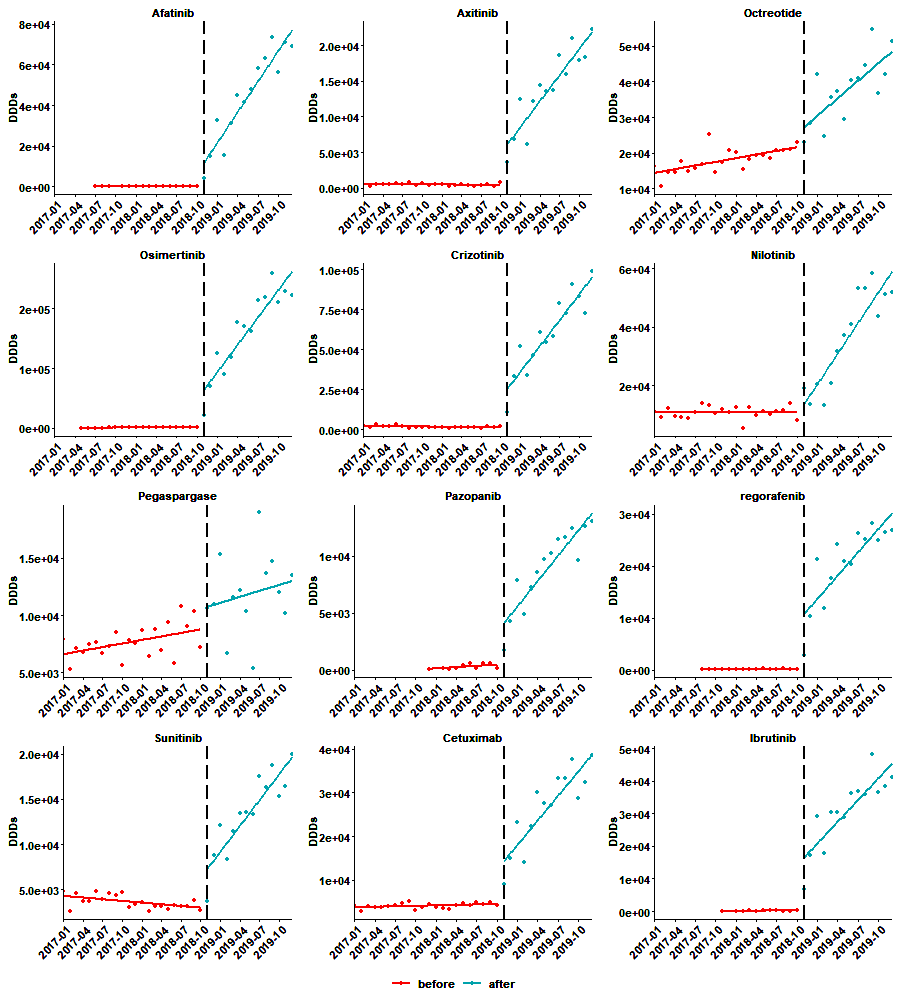


**Figure S2. Trend in monthly DDDs of 12 anticancer drug from January 2017 to December 2019.**
